# Glutamine Synthetase 1 Functions in Spermatogenesis in the Silkworm, *Bombyx mori*

**DOI:** 10.3390/insects17020135

**Published:** 2026-01-24

**Authors:** Zelin Fan, Lulu Zhang, Surui Zhang, Jiayin Zhang, Cuiqi Fang, Xiuping Lu, Xingfu Zha

**Affiliations:** State Key Laboratory of Resource Insects, College of Sericulture, Textile and Biomass Sciences, Southwest University, Chongqing 400715, China; fzl13294@163.com (Z.F.); 13752804482@163.com (L.Z.); 13668261755@163.com (S.Z.); 15981139421@163.com (J.Z.); fcq13426554841@163.com (C.F.); xiupinglulu@163.com (X.L.)

**Keywords:** BmGS1, *Bombyx mori*, virtual screening, inhibitors, male sterility

## Abstract

Glutamine synthetase, a critical enzyme catalyzing the conversion of glutamate and ammonia into glutamine, has been shown to influence sperm development in mammals. Here, we carried out functional analysis of *Bombyx mori* homolog of glutamine synthetase 1 (*BmGS1*) and screened its small-molecule inhibitor. RT-PCR and qPCR showed that *BmGS1* was specifically expressed in the testis of the silkworm, with the highest expression in the moth stage. Subcellular localization revealed that the BmGS1 protein was localized in mitochondria and cytoplasm. Identification of upstream regulatory factors revealed that the expression of *BmGS1* is positively regulated by the sex-related transcription factor *Bmdsx*. Virtual screening, molecular docking and MD simulations showed that the small molecule Ethylhexyl triazone (ET), as well as the known GS inhibitor L-Methionine -DL-sulfoximine (MSX), could be stably bound to BmGS1. Subsequently, site-specific mutation and fluorescence binding assays revealed that the putative key sites of ET binding to the protein were E79 and R265, and the putative key sites of MSX binding to the protein were E81, R245, and R286. Both in vitro and in vivo experiments demonstrated that inhibitor treatment significantly attenuated BmGS1 enzymatic activity. Inhibitor-injected silkworms showed reduced fertilization rates compared to control groups. Our findings raise BmGS1 as a potential target for silkworm sterility.

## Simple Summary

While glutamine synthetase (GS) has been documented in mammalian spermatogenesis, its functional role in insect male reproductive systems remains largely unexplored. In this study, we investigated *Bombyx mori GS1* (*BmGS1*) and demonstrated its testis-specific expression pattern, with peak expression observed during the moth stage. Subcellular localization analysis revealed its dual distribution in both mitochondria and cytoplasm. Investigation into its upstream regulatory factors demonstrated that the expression of *BmGS1* is positively regulated by the reproduction-associated transcription factor *Bmdsx*. Through virtual screening and molecular dynamics simulation, two small-molecule inhibitors, ET and MSX, which can stably bind to the BmGS1 protein were identified. Enzymatic activity assays and fluorescence binding experiments verified the specific binding and inhibitory effects of the two small molecules on BmGS1. Experiments showed that injection of the inhibitors could reduce the fertilization rate of silkworms. This study provides a valuable clue and potential inhibitors for pest sterility technology.

## 1. Introduction

Insect infertility techniques involve the introduction of sterile male insects to reduce pest reproduction, thereby providing an environmentally sustainable approach to pest control [1,2]. This method is widely applied, especially in the control of fruit flies, mosquitoes, and other pests [3,4]. With the continuous expansion of its application, genes involved in reproductive regulation—particularly key genes participating in spermatogenesis—have emerged as critical targets for breeding sterile male insects. Insect sterility technology holds promise for evolving into a core component of next-generation integrated pest management strategies.

The silkworm represents an established model organism for Lepidopteran studies. Multiple testis-enriched genes associated with spermatogenesis have been identified in this species, including *BmPMFBP1*, *BmSer1*, and *BmC/EBPZ*, among others [5,6,7]. Comprehensive transcriptome analysis of the testis of the silkworm reveals the expression of 1705 testis-specific genes [8]. Additionally, genes encoding serine/threonine protein kinases, organic cation transporter proteins, tyrosine protein kinases, lncRNAs, and immune-related proteins have been identified as crucial contributors to spermatogenesis and testicular development [9]. During fertilization, sperm motility serves as a crucial functional parameter, specifically quantified as the percentage of spermatozoa exhibiting progressive linear motility relative to the total sperm count, which significantly impacts fertilization success rates [10]. Genetic defects in specific genes and various non-biological factors can impair sperm motility, potentially leading to fertility disorders [11,12].

Glutamine synthetases (GSs) are enzymes that catalyze the ATP-dependent conversion of glutamate and ammonia to glutamine, exhibiting tissue-specific and developmentally regulated expression patterns [13]. In plants, GSs have been extensively studied, particularly for their roles in maintaining nitrogen flow, facilitating internal nitrogen sensing during critical developmental stages, and enhancing nitrogen-use efficiency in crops [14]. In insects, GS participates in various physiological processes. Studies in *Drosophila melanogaster* have shown that GS enhances neuronal survival, while research in *Bactrocera dorsalis* has demonstrated its involvement in ovarian development through *GS* gene knockdown experiments [15,16]. Current research on GS in reproductive systems remains limited. Immunofluorescence studies have localized GS to the head of human spermatozoa. Additionally, glutamine, a component of seminal plasma, has been demonstrated to influence mammalian sperm viability [17]. Studies demonstrate that GS exhibits significantly higher enzymatic activity in the caput epididymis compared to other tissues in mice. This elevated activity plays a crucial role in maintaining the optimal microenvironment for sperm maturation by eliminating glutamate and ammonia, thereby enhancing sperm motility, restoring acid–base balance, and ultimately promoting spermatogenesis [18,19]. In *Bombyx mori,* two distinct forms of GS isozymes have been identified: the mitochondrial GS1 and the cytoplasmic GS2. The GS1 isozyme consists of a single subtype and exhibits highly tissue-specific expression patterns, whereas the GS2 isozyme comprises 2 to 4 subtypes.

Virtual screening, a computational approach for identifying small-molecule ligands based on the structures of target proteins and ligands, has been instrumental in the discovery of small-molecule inhibitors for therapeutic targets [20,21]. The use of virtual screening techniques to predict possible potential drugs plays an important role in identifying inhibitors targeting protease active sites. Molecular dynamics (MD) simulation holds significant value in molecular biology and drug discovery, serving as an effective method to predict ligand–target interactions while accounting for target flexibility [22]. As a key enzyme in the glutamine metabolic pathway, inhibition of GS1 may compromise the functional integrity of sperm, leading to reduced motility. In this study, we utilized MD and virtual screening to identify potential inhibitors of glutamine synthetase 1 (BmGS1) in the silkworm, *Bombyx mori*, and explored its role as a target for inducing male sterility.

## 2. Materials and Methods

### 2.1. Insects and Cells

The silkworms (D9L) were provided by the State Key Laboratory of Resource Insects (Southwest University, Chongqing, China) and were routinely bred with a diet of mulberry leaves at 25 ± 1 °C with 70 ± 5% humidity. The cell line used in this study was the *Bombyx mori* embryonic cell line (BmE), which was preserved by the State Key Laboratory of Silkworm Genome Biology and cultured in Grace Medium supplemented with 10% fetal bovine serum (FBS, Gibco, Waltham, MA, USA) and 200,000 units of penicillin–streptomycin solution per liter. During the culture process, the cells were passaged approximately every 4 days, depending on their growth status, and maintained in a constant temperature incubator at 27 °C.

### 2.2. Real-Time Quantitative PCR

The silkworms were reared until the third day of the fifth instar, and the gonads, head, epidermis, silk glands, midgut, and fat body tissues were taken from males and females, respectively. We selected the testis of male silkworms from the first (L5D1), third (L5D3), fifth (L5D5), and seventh (L5D7) days of the fifth instar; wandering stage (W1); pre-pupae stage (PP); first (P1), third (P3), fifth (P5), seventh (P7), and ninth (P9) days of the pupae stage; and moth stage (M), and males with the same state of growth in P3. The testis were removed, and the contents and outer membrane were separated from the testis. The tissues were washed in PBS, blotted dry with high-pressure filter paper, collected into RNase-free 1.5 mL centrifuge tubes, and stored at −80 °C after liquid nitrogen flash-freezing. RNA was extracted using TRIZOL reagent (Invitrogen, Carlsbad, CA, USA) and reverse-transcribed according to the instructions of PrimeScriptTM RT reagent Kit (TaKaRa, Otsu, Japan). Quantitative analysis was performed with the Novo Start SYBR Qpcr SuperMix Plus kit (Invitrogen, Carlsbad, CA, USA). The silkworm *sw* gene was used as the internal reference. qPCR was performed using an ABI 7500 Real-Time PCR System with the default thermal cycling protocol. Gene expression levels were calculated using the 2^−ΔΔCT^ method, and data visualization was conducted using GraphPad Prism 8.0. Three biological replicates and three technical replicates were conducted for each qPCR run.

### 2.3. Semi-Quantitative PCR

Specific primers were designed based on the coding sequence of the *BmGS1* gene using Primer Premier 5 software. Similarly, specific primers for the *BmActin3* reference gene were designed. PCR amplification was performed using 200 ng/μL cDNA as a template. Subsequently, normalized cDNA from various tissues was used as a template for PCR amplification, followed by nucleic acid electrophoresis analysis. All primer sequences are detailed in Table S1.

### 2.4. Subcellular Localization

When the cell density reached 70%, the cells were seeded in cell-well plates and transfected with either pSL1180-BmGS1 or a control plasmid. After 48 h, the medium was discarded, and the cells were washed three times with PBS. The cells were then stained using the Mitochondrial Membrane Potential Detection Kit (Beyotime, Shanghai, China) in an incubator at 27 °C for 15 min, followed by another three washes with PBS. The cells were observed and photographed using the laboratory EVOS FL automated imaging system, with a scale bar of 10 µm.

### 2.5. Dual-Luciferase Reporter Experiment

Information related to the *BmGS1* gene was retrieved from the NCBI database, and its promoter region was localized in GenBank. In this study, a fragment spanning from 2000 bp upstream to 100 bp downstream of the translation initiation site of this gene was selected as the promoter sequence for subsequent functional verification experiments. Potential transcription factor binding sites within the promoter sequence were predicted and analyzed using the JASPAR online platform (https://jaspar.genereg.net/ accessed on 21 January 2026). The genes *dsx* and *abd-A*, which are closely associated with reproductive functions in *Drosophila*, were screened out as candidate targets for further research to explore their roles in regulatory mechanisms.

The *BmGS1* promoter fragment was cloned into the dual-luciferase reporter vector pGL3. When BmE cells grew to an appropriate density, they were seeded in 24-well culture plates. The transcription factor expression vectors (pSL-1180-Bmdsx or pSL-1180-Bmabd-A), the promoter-pGL3 reporter vector, and the internal reference plasmid were co-transfected into the cells. After 48 h, we tested the fluorescence activity with a dual-luciferase reporter kit (Promega, Madison, WI, USA).

### 2.6. Electrophoretic Mobility Shift Assay (EMSA) Binding Experiment

EMSA can be used to verify whether transcription factors interact with promoters. In this study, we first purified Bmdsx and Bmabd-A proteins, and then synthesized biotin-labeled probes based on the predicted binding sites. The binding of protein and nucleic acid was verified with a chemiluminescence kit (Beyotime, Shanghai, China).

### 2.7. Target Protein Acquisition and Preprocessing

The protein sequence of BmGS1, was used for template searches in SWISS-MODEL (https://swissmodel.expasy.org/ accessed on 21 January 2026). Templates with closely related enzyme species, high sequence homology, and comprehensive sequence coverage were selected. Homology modeling was subsequently conducted based on GMQE and QMEAN scores. After constructing the homology model, its structural rationality was evaluated using SAVES v6.0 (https://saves.mbi.ucla.edu/ accessed on 21 January 2026).

The BmGS1 protein activity pockets were predicted using DoGSiteScorer (https://proteins.Plus/ accessed on 21 January 2026) and ranked based on their size, surface area, and druggability score. Chimera software (1.18) was used to visualize the predicted active pockets of BmGS1. The top three ranked pockets were selected and displayed along with the BmGS1 structure.

Proteins were prepared for virtual screening and molecular docking by hydrogenation, charge assignment, and removal of water molecules using AutoDockTools-1.5.7.

### 2.8. Establishment of a Small-Molecule Database

Small-molecule compounds for virtual screening were obtained from the PubChem database (https://pubchem.ncbi.nlm.nih.gov accessed on 21 January 2026). The FDA Global Substance Registry library was selected, resulting in a dataset of 5764 three-dimensional small-molecule structures. OpenBabel software (2.4.1) was used for the conformational conversion of the compounds. Additionally, the literature reports that L-Methionine-DL-sulfoximine (MSX) is a specific inhibitor of glutamine synthetase, widely applied in medicine, pharmacology, and biology [23,24]. We therefore selected it as one of the candidate small molecules.

### 2.9. Virtual Screening and Molecular Docking

The preprocessed small molecules were subjected to batch docking using a screening software. The first active pocket position predicted by DoGSiteScorer was selected, and the top-ranked small molecules in terms of affinity were obtained. Then, AutoDock Vina was used for molecular docking, and the vina docking file config was exported. AutoDock Vina was run, and relevant studies indicate that the more negative the binding energy, the stronger the binding [25,26]. Therefore, by considering both binding energy and practicality, small molecules with a free binding energy lower than −4.0 kcal/mol and reasonable binding conformations were selected as potential binders of BmGS1.

### 2.10. Molecular Dynamics Simulations

To investigate the binding modes between small molecules and BmGS1, 100 ns MD simulations were conducted to explore their interactions. The protein and optimal ligands were separated from the docking results, and ligand force field files were generated using the Antechamber tool in AmberTools and converted to GROMACS-compatible formats using the ACPYPE tool. The General Amber Force Field (GAFF) was applied to the ligands, while the AMBER14SB force field and TIP3P water model were used for the protein. The protein and ligand files were merged to construct the simulation system. MD simulations were performed using GROMACS under periodic boundary conditions at constant temperature (298 K) and pressure (1 bar). Bonds involving hydrogen atoms were constrained with the LINCS algorithm, using a 2 fs integration time step. Electrostatic interactions were calculated with the Particle-Mesh Ewald (PME) method (cutoff distance: 1.2 nm), and the non-bonded interaction cutoff was set to 10 Å, updated every 10 steps. System equilibration was carried out with 100 ps of NVT and NPT simulations, followed by 100 ns MD simulations with snapshots saved every 10 ps. Simulation trajectories were analyzed using VMD (1.9.3) and PyMOL (3.0.4), and binding free energy was calculated using the g_mmpbsa tool based on the Molecular Mechanics Poisson Boltzmann Surface Area (MMPBSA) method.

### 2.11. Key Amino Acid Mutations and In Vitro Fluorescence Binding Validation

To confirm the binding of the two screened ligand molecules to BmGS1 and to investigate the roles of the putative key amino acids involved in this interaction, we performed targeted mutagenesis at positions Glu79, Arg265, Glu81, Arg245, and Arg286 based on molecular dynamics simulations. A Pcold-TF-BmGS1 prokaryotic expression vector was constructed for protein purification. The binding affinity was measured using a fluorescence binding assay, where the fluorescence intensity displayed a linear relationship with ligand concentration, allowing the determination of the dissociation constant (Kd). Additionally, the enzymatic activity of BmGS1 was assessed after the addition of the small molecules using the Glutamine Synthetase Activity Assay Kit (Boxbio, Beijing, China) to verify whether the ligands affected the protein’s activity.

### 2.12. Injections of Inhibitors into Silkworms

The results showed that the intensity of the protein absorption peak was significantly reduced upon the addition of 2 μM small molecules, indicating the optimal binding efficiency. Therefore, 2 μM of the small molecule was used for injection. Each group included three replicates, with 10 male silkworms in each group. And the testes were collected at 12 h, 24 h, and 48 h post-treatment for the measurement of glutamine synthetase enzymatic activity.

During the pupal stage, a timed injection protocol was initiated on the first day of pupation. Male silkworms with similar size and body weight were selected for injection. A total of three groups, each consisting of 15 individuals, received injections every 48 h. The inhibitors ET and MSX were dissolved in 0.1% DMSO to a final concentration of 2 μM. Each injection, with a volume of 2 µL, was administered into the testes of male silkworm pupae from the dorsal side of the fifth abdominal segment. Males that successfully molted to the adult stage were subjected to mating trials with untreated females, as well as with males treated with each of the two small molecules. After oviposition, the number of eggs laid and the number of fertilized eggs were recorded for 15 brood cycles in each treatment group to calculate fertilization rates.

## 3. Results

### 3.1. BmGS1 Predominantly Expressed in Testis of Silkworm

**Figure 1:**
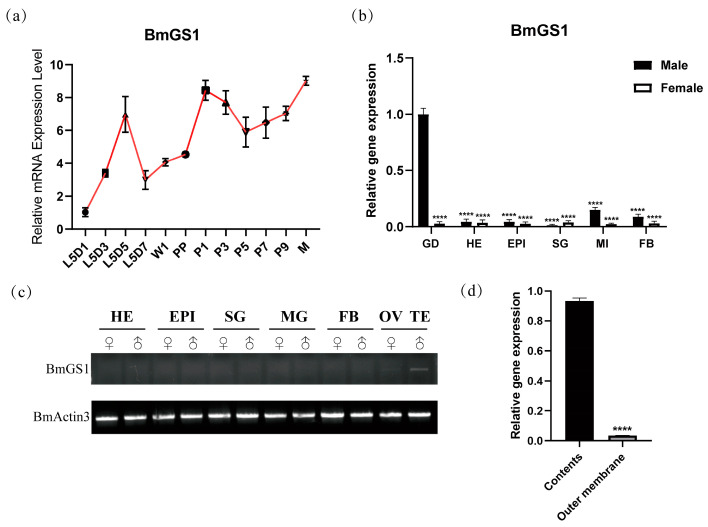
Expression pattern of the *BmGS1* gene. (**a**) Period expression of *BmGS1*; L5D1: first day of the fifth instar; L5D3: third day of the fifth instar; L5D5: fifth day of the fifth instar; L5D7: seventh day of the fifth instar; W1: wandering period; PP: pre-pupae period; P1: first day of pupae; P3: third day of pupae; P5: fifth day of pupae; P7: seventh day of pupae; P9: ninth day of pupae; M: moth period. (**b**) qPCR tissue expression. (**c**) Semi-quantitative analysis of *BmGS1* gene expression in each tissue of the third day of the fifth instar silkworms. *BmActin3* is an internal reference gene. GD, gonad; TE, testis; OV, ovary; SG, silk gland; FB, fat body; EPI, epidermis; MI/MG, midgut; HE, head. (**d**) Testicular contents and outer membrane expression of *BmGS1*. Three groups were repeated to take the mean, and the control group mean was used as the normalization factor; *n* = 3 independent experiments; *t*-test—**** indicates significance level *p* < 0.0001.

To investigate the spatio-temporal expression pattern of the *BmGS1* gene, RT-PCR and qPCR were conducted to analyze its expression in various tissues of male and female silkworms, including the gonads, head, epidermis, silk glands, midgut, and fat body. RT-PCR and qPCR revealed that *BmGS1* was predominantly expressed in testicular tissues (Figure 1b,c), with high expression observed in the testis contents (Figure 1d). To further elucidate the temporal expression pattern of *BmGS1* in testicular tissues, qPCR analysis demonstrated that this gene was expressed throughout the fifth instar larval, pupal, and moth stages (Figure 1a), suggesting its functional involvement in both spermatogenesis and sperm maturation.

**Figure 2:**
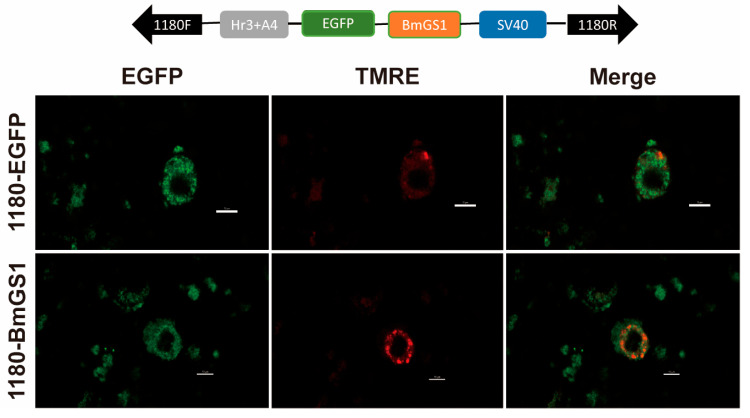
Cellular location of the BmGS1. Subcellular location of BmGS1 in the silkworm cell lines (BmE) and pSL1180-EGFP treated cells as a control. Red fluorescence for TMER-treated mitochondria, and merged images are shown (Scale bar: 10 µm).

To investigate cellular expression, a recombinant vector, pSL1180-BmGS1, was constructed for overexpression in silkworm cell lines. Subcellular localization experiments revealed that BmGS1 is expressed in the mitochondria and cytoplasm (Figure 2).

### 3.2. Sex-Related Transcription Factor Bmdsx Positively Regulates BmGS1

To investigate the upstream regulatory factors of the *BmGS1* gene, we analyzed its promoter region and cloned the promoter fragment into the dual-luciferase reporter vector pGL3 to detect its transcriptional activity. Transcription factors that potentially bind to the *BmGS1* promoter were predicted using the JASPAR database. Considering the high expression of *BmGS1* in the testis of *Bombyx mori*, we focused on the predicted transcription factors *Bmdsx* and *Bmabd-A*. To explore the regulatory effects of these two transcription factors on *BmGS1*, we constructed the expression vectors pSL-1180-Bmdsx and pSL-1180-Bmabd-A.

**Figure 3:**
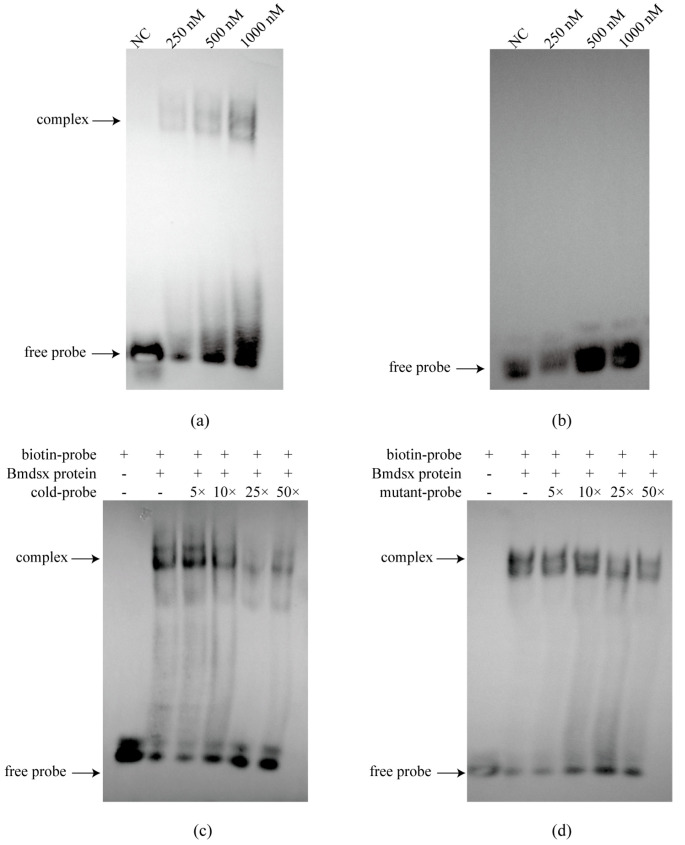
The binding of the transcription factor Bmdsx/Bmabd-A and *BmGS1* promoter. (**a**) Combination experiment of the *BmGS1* promoter with Bmdsx. (**b**) Combination experiment of the *BmGS1* promoter with Bmabd-A. (**c**) EMSA cold competition experiment with the transcription factor Bmdsx and *BmGS1* promoter. (**d**) EMSA cold competition experiments for mutant probes with the transcription factor Bmdsx and *BmGS1* promoter.

**Figure 4:**
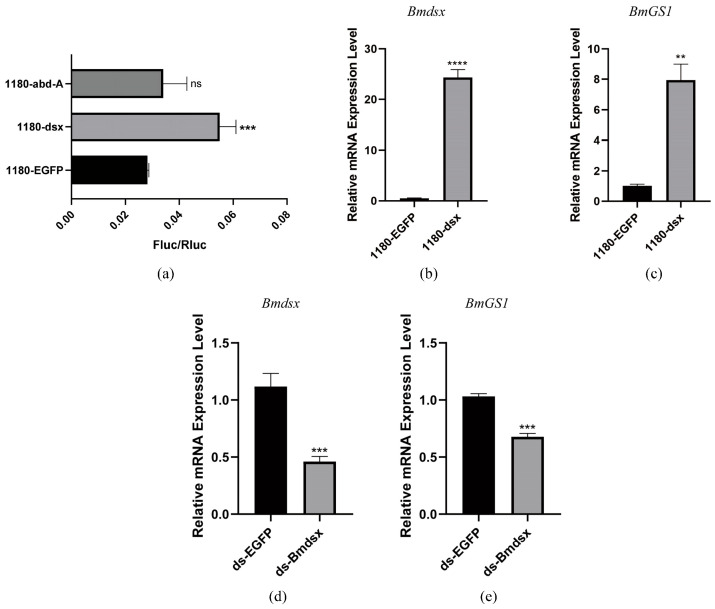
The regulatory effect of the transcription factor *Bmdsx* on *BmGS1*. (**a**) Dual-luciferase reporter experiment on the transcription factor Bmdsx/Bmabd-A and *BmGS1* promoter. (**b,c**) Detection of *BmGS1* expression after overexpression of *Bmdsx* at the cellular level. (**d,e**) Detection of *BmGS1* expression levels after individual-level interference with *Bmdsx*. Three groups were repeated to take the mean, and the control group mean was used as the normalization factor; *n* = 3 independent experiments; *t*-test—ns means no significant difference, ** indicates significance level *p* < 0.01, *** indicates significance level *p* < 0.001, **** indicates significance level *p* < 0.0001.

To verify the specificity of the interaction between transcription factors and the promoter, we purified Bmdsx and Bmabd-A proteins and performed EMSA. The results showed that Bmdsx was able to bind to the *BmGS1* promoter (Figure 3a). Cold competition experiments and mutant probe experiments further confirmed the specific binding of Bmdsx to the *BmGS1* promoter (Figure 3c,d). In contrast, Bmabd-A did not exhibit any binding bands, indicating that it has no binding activity with the *BmGS1* promoter (Figure 3b). Dual-luciferase activity assays revealed that *Bmdsx* promotes the expression of *BmGS1*, whereas *Bmabd-A* exhibits no significant regulatory effect on *BmGS1* (Figure 4a).

To further confirm whether *Bmdsx* regulates *BmGS1*, we detected the expression change in *BmGS1* after overexpressing *Bmdsx* at the cellular level, and found that the expression level of *BmGS1* was upregulated with the overexpression of *Bmdsx* (Figure 4b,c). At the individual level, after interfering with *Bmdsx*, we detected the expression change in *BmGS1* and observed that the expression level of *BmGS1* was downregulated following the interference of *Bmdsx* (Figure 4d,e). These results further supported the positive regulatory effect of *Bmdsx* on *BmGS1*.

### 3.3. Protein Structure of BmGS1 Predicted

**Figure 5:**
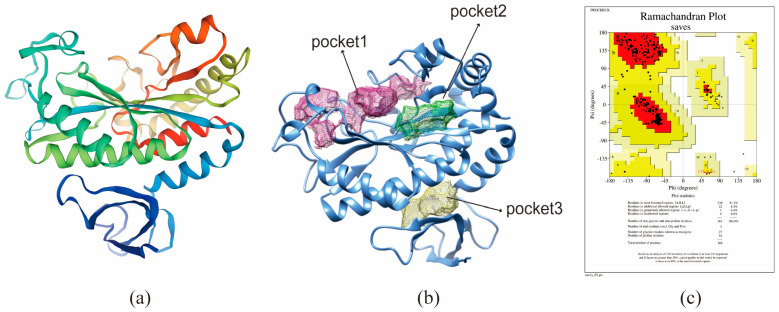
Homology modeling and combined pocket prediction analysis map. (**a**) 3D structure of BmGS1 protein. (**b**) Analysis of protein binding pocket. (**c**) 3D structural Lagrangian of protein.

The BmGS1 protein sequence was submitted to SWISS-MODEL for protein modeling (template PDB: 8DNU.A; GMQE score: 0.79), and models with high sequence identity were selected for further analysis (Figure 5a). Structural validation using the ProCheck tool on SAVESv6.0 revealed that over 99% of residues were in fully allowed or allowed regions in the Ramachandran plot, indicating a reliable protein structure for subsequent studies (Figure 5c). Ramachandran plot statistics are provided in Supplementary Table S2.

The saved protein structure was submitted to DoGSiteScorer (https://proteins.plus/ accessed on 21 January 2026) for the prediction of potential binding pockets. Seven predicted pockets were ranked based on size, surface area, and druggability scores. The top-ranked pocket (Pocket 1) was selected as the preferred binding site for inhibitors (Figure 5b).

### 3.4. Small Molecules MSX and ET Were Candidate Inhibitors of BmGS1 by Virtual Screening and Molecular Docking

**Figure 6:**
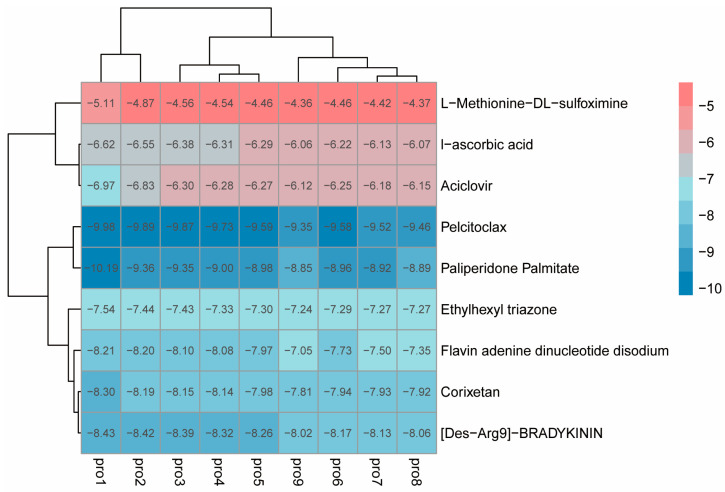
Heatmap of binding energy between protein and small molecules.

The preprocessed small molecules were subjected to batch docking using screening software, and several small molecules with higher affinity rankings were selected. Ultimately, eight small molecules from the molecular library were chosen (docking scores shown in Table S3), and L-Methionine-DL-sulfoximine (MSX), a small molecule reported to inhibit glutamine synthetase activity in other species, was also selected as a candidate inhibitor. Then, the local software AutoDock Vina was used to perform molecular docking on these nine candidate small molecules. Small molecules with free binding energy lower than −4.0 kcal/mol and reasonable site conformations were output, and a binding energy heatmap was drawn. Based on the binding energy of the small molecules and their commercial availability, two ligand small molecules were finally selected, namely Ethylhexyl triazone (ET) and MSX (TargetMol, Shanghai, China) (Figure 6).

### 3.5. MSX and ET Capable to Bind to BmGS1 by Analysis of Molecular Dynamics

#### 3.5.1. Stable Binding of Small Molecules to BmGS1

**Figure 7:**
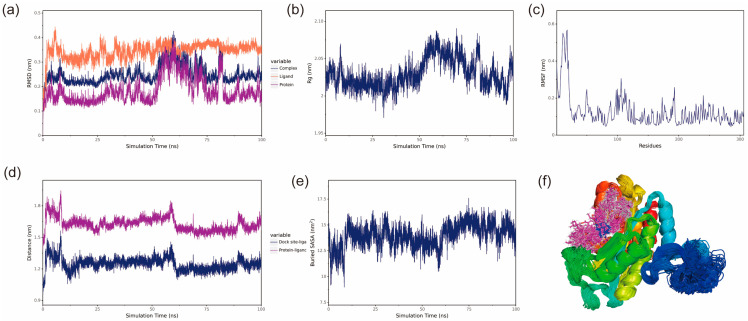
BmGS1-ET stability analysis. (**a**) RMSD of the complex, protein and small-molecule ligand; (**b**) Rg of the complex; (**c**) RMSF of the protein in the complex; (**d**) protein and small-molecule binding site spacing (dock site–ligand); (**e**) encapsulation area between small molecule and protein (Buried SASA); (**f**) simulated conformation.

**Figure 8:**
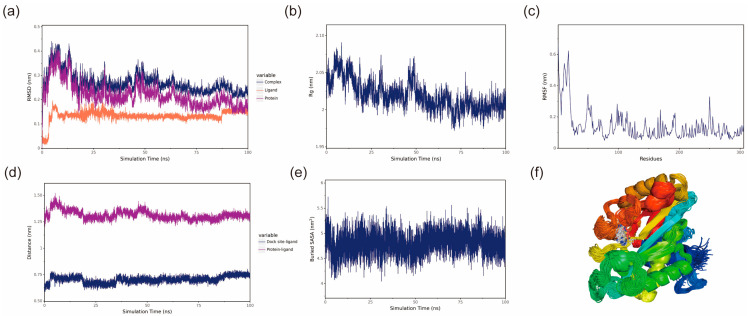
BmGS1-MSX stability analysis. (**a**) RMSD of the complex, protein and small-molecule ligand; (**b**) Rg of the complex; (**c**) RMSF of the protein in the complex; (**d**) protein and small-molecule binding site spacing (dock site–ligand); (**e**) encapsulation area between small molecule and protein (Buried SASA); (**f**) simulated conformation.

Root mean square deviation (RMSD) represents the sum of all atomic deviations of the conformation from the target conformation at a given moment, and it is an important measure of the stability of a system. The RMSD values of the protein and protein–ligand complexes fluctuate synchronously, suggesting that changes in the complex RMSD are primarily driven by the protein. Both the BmGS1-ET and BmGS1-MSX complexes showed fluctuations between 50 and 70 ns but gradually stabilized, indicating that the protein structure remained stable. The small molecules maintained stable RMSD values, confirming their conformational stability (Figure 7a and Figure 8a).

The Radius of Gyration (Rg) can be used to describe the overall structural changes and can be used to characterize the compactness of the protein structure, with larger changes in Rg indicating a more swollen system [27]. The Rg of the BmGS1-ET complexes fluctuated during 50–70 ns, primarily due to changes in the protein structure, as small molecules have a negligible influence on Rg. After 70 ns, Rg fluctuations decreased, indicating that the complexes stabilized over time. The Rg of BmGS1-MSX gradually decreases and stabilizes, which indicates that the complex is gradually stabilized (Figure 7b and Figure 8b).

Root mean square fluctuation (RMSF) refers to the rise and fall of an atom relative to its average position, reflecting the stability or flexibility of the protein structure and the root mean square displacement of each amino acid residue in the protein [28]. High RMSF values indicate flexible regions or functional sites, while low RMSF values suggest stable residues, often located within the protein core or structural domains. Most residues in the protein were stable, with low RMSF values, indicating a well-conserved structural framework (Figure 7c and Figure 8c).

The center of mass evolution analysis is performed to analyze the state of small molecules on the surface of the protein, to obtain the initial docking site of the small molecule, and to analyze the distance between the center of mass of the residues of the initial docking site and the center of mass of the small molecule. The distance between the small molecule and the protein heart is also analyzed, and by analyzing these distances, the binding state of the small molecule to the protein is obtained. The analysis results show that the distances between the centroids of the two small molecules, ET and MSX, and the BmGS1 protein, as well as the relative positions of the ligands to the initial binding sites, remain relatively stable without significant fluctuations. This result indicates that throughout the simulation process, the ligand molecules are stably bound to the initial recognition sites of the protein, suggesting a stable binding between the small molecules and the protein (Figure 7d and Figure 8d).

The Buried SASA of the small molecules embedded in the protein can reflect the size of the binding interface of the small molecules bound to the protein, and the binding status of the small molecules and the protein can be determined by analyzing the Buried SASA. The analysis revealed a stepwise increase in Buried SASA during the simulation, indicating a gradual expansion of the contact area between the two small molecules (ET and MSX) and the BmGS1 protein, suggesting progressive stabilization of their binding (Figure 7e and Figure 8e).

Structural alignment of the simulated conformations of ET and MSX with BmGS1 revealed that both small molecules in the BmGS1-ET and BmGS1-MSX complexes maintained positions near their initial binding sites with excellent conformational overlap (Figure 7f and Figure 8f). These findings demonstrate stable binding of the ligands to the protein throughout the simulation period.

#### 3.5.2. Small Molecule–Protein Hydrogen Bonding Interactions

**Figure 9:**
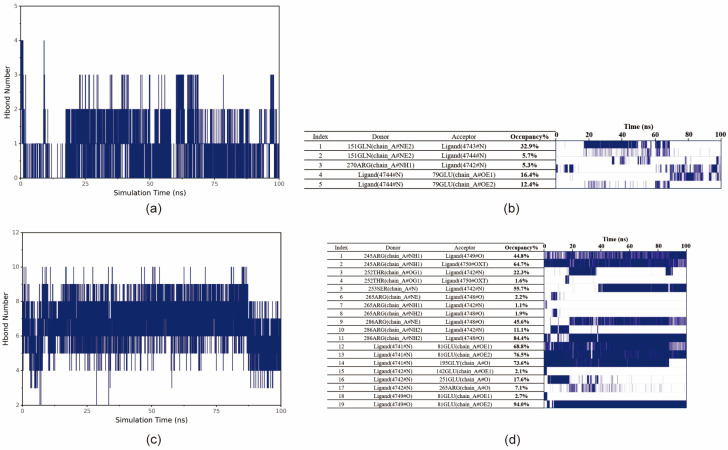
Analysis of small molecule–protein hydrogen bonding interactions. (**a**) Number of hydrogen bonds formed between ET and BmGS1. (**b**) Frequency of hydrogen bonding between ET and BmGS1. Acceptor, donor, and occupancy of hydrogen bonding pairs on the left side; on the right side, the frequency of formation of the corresponding hydrogen bond. The density of the line represents the frequency of hydrogen bond formation. (**c**) Number of hydrogen bonds formed between MSX and BmGS1. (**d**) Frequency of hydrogen bonding between MSX and BmGS1.

Hydrogen bonding interactions are an important force in protein–ligand binding. Hydrogen bonds are related to electrostatic interactions and can reflect the strength of electrostatic interactions. As can be seen from the figure, the number of hydrogen bonds between ET and the protein remains stable, mainly fluctuating between 0 and 2 (Figure 9a). The number of hydrogen bonds between MSX and the protein is higher and remains stable, mainly fluctuating between 4 and 9 (Figure 9c).

In order to obtain further information about the residues that form hydrogen bonds with small molecules in proteins and the stability of hydrogen bonds, the occupancy of hydrogen bonds formed between small molecules and proteins (frequency of hydrogen bond formation) was analyzed. The acceptor, donor, and occupancy of hydrogen bonding pairs and the corresponding hydrogen bonding frequency are shown in the figure, and the density of the lines represents the frequency of hydrogen bonding. The frequency of hydrogen bonding between ET and protein changes more significantly around 20 ns and 70 ns; for example, the stability of ET:79GLU increases after 70 ns, which is caused by the change in the small molecule–protein binding conformation (Figure 9b). MSX forms some of the more stable residues with the protein, and some of the more stable residues are found in the small molecule–protein pairs. MSX formed some of the more stable hydrogen bonds with the protein, such as 245ARG:MSX and 286ARG:MSX (Figure 9d).

#### 3.5.3. Interaction Between Small Molecules and Proteins

**Figure 10:**
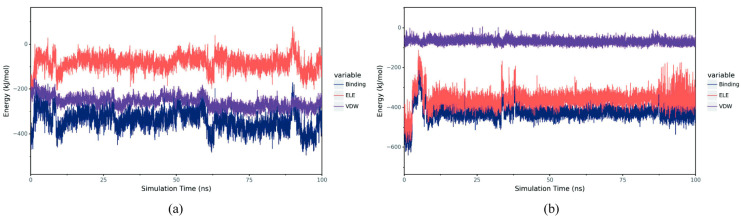
Binding energy between small molecules and proteins, including VDW and ELE. (**a**) Binding energy between ET and BmGS1. (**b**) Binding energy between MSX and BmGS1.

VDW represents van der Waals forces and hydrophobic interactions, while ELE represents electrostatic interactions. By summing up the energy contributions of VDW and ELE, the binding free energy (Binding energy) of the system can be obtained. Under the condition of ignoring the solvation effect, this value can characterize the binding strength between the ligand and the target protein. The results show that in the complex BmGS1-ET, VDW is more stable relative to ELE. Although ELE fluctuates relatively greatly, it remains stable as a whole, indicating that the binding between ET and BmGS1 is stable. In the complex BmGS1-MSX, both VDW and ELE are very stable, which also indicates that the binding between MSX and BmGS1 is extremely stable (Figure 10).

**Figure 11:**
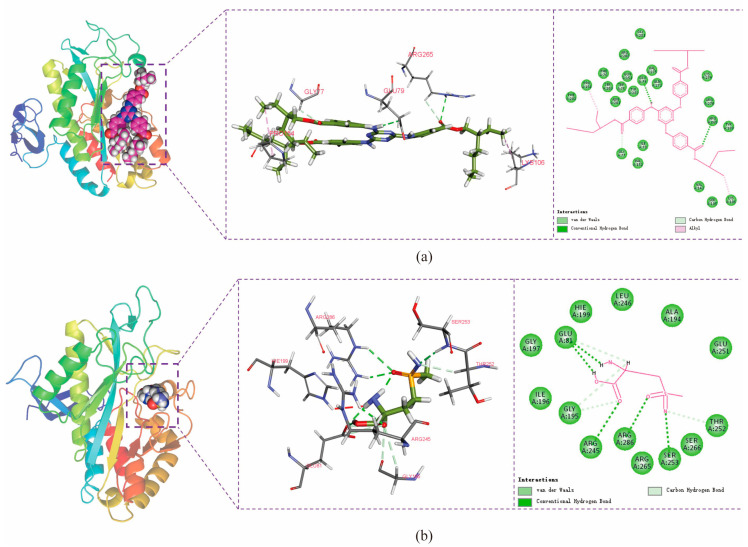
Protein–small-molecule interaction. (**a**) The interaction of ET with BmGS1. (**b**) The interaction of MSX with BmGS1. The 3D structure of the BmGS1 protein is shown on the left side, while the 3D and 2D structures of small molecules are shown on the right side in sequence. Different bond colors represent different forces of protein formation.

The conformation at the end of the simulation was selected and its structure and interactions were analyzed using Discovery Studio Visualizer software (2025). The amino acids GLU79 and ARG265 in the protein formed hydrogen-bonding interactions with ET, PRO154 and LYS106 formed alkyl-hydrophobic interactions with ET, and amino acids VAL152 and TYR109 formed van der Waals force interactions with ET (Figure 11a). GLU81, ARG245, ARG286, and SER253 form hydrogen bonds with MSX, while GLU81, GLY195, and THR252 form carbon hydrogen bonding interactions (weaker hydrogen bonding) with MSX, and amino acids such as ILE196 and GLU251 form van der Waals force interactions with MSX (Figure 11b). The quantitative energy contributions of the interactions involving these residues are provided in Supplementary Table S4.

### 3.6. Mutation of Key Sites of BmGS1 Protein Affected Binding Activity

The small-molecule and protein concentrations were adjusted to 2 µM. Without the small molecule, the BmGS1 protein displayed a strong absorption peak at 346–347 nm. After adding the small molecule, the absorption peak decreased, indicating binding between the protein and the small molecule. The optimal binding condition was observed with 2 µM of both the protein and the small molecule.

**Figure 12:**
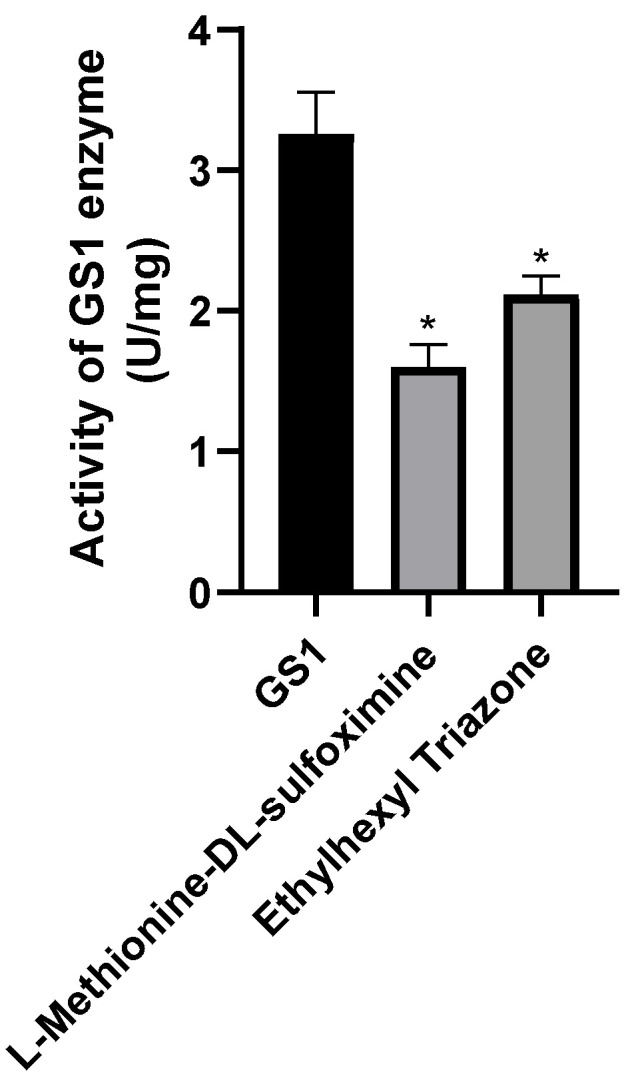
Determination of in vitro binding enzyme activity of proteins and small molecules. Three groups were repeated to take the mean, and the control group mean was used as the normalization factor; *n* = 3 independent experiments; *t*-test, * indicates significance level *p* < 0.05.

We measured the enzyme activity of the proteins after small-molecule treatment and found that both ET and MSX significantly reduced glutamine synthetase activity compared to untreated proteins (Figure 12).

**Figure 13:**
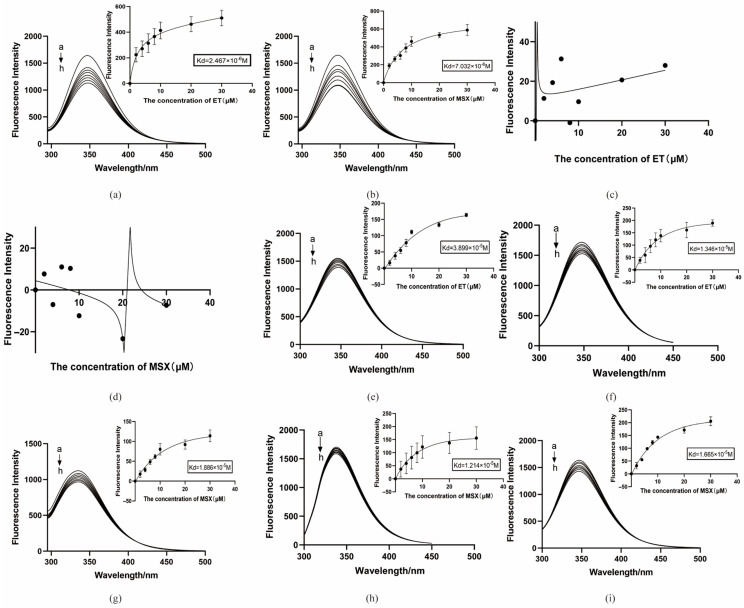
Protein binding to small molecules in vitro. (**a**) ET binds to BmGS1 protein; (**b**) MSX binds to BmGS1 protein; (**c**) ET binds to pCOLD-TF; (**d**) MSX binds to pCOLD-TF; (**e**) ET binds to BmGS1-E79A mut protein; (**f**) ET binds to BmGS1-R265A mut protein; (**g**) MSX binds to BmGS1-R245A mut protein; (**h**) MSX binds to BmGS1-E81A mut protein; (**i**) MSX binds to BmGS1-R286A mut protein. a→h is the concentration of small molecules added by small molecules, in the order 0 µM, 2 µM, 4 µM, 6 µM, 8 µM, 10 µM, 20 µM, and 30 µM. The curve was fitted by nonlinear regression using the “One site—Total” model. Data points represent the mean of three independent binding experiments (*n* = 3 independent experiments). The R^2^ values for the curves are provided in Supplementary Table S5.

In vitro binding experiments revealed dissociation constants (Kd) of 2.467 × 10^−6^ M for ET (Figure 13a) and 7.032 × 10^−6^ M for MSX (Figure 13b). Both values, being below 10 µM, indicate strong binding, with ET showing a higher binding efficiency than MSX. In contrast, the empty pCOLD-TF protein did not show stable peak changes, suggesting no binding to the small molecules (Figure 13c,d).

Based on molecular dynamics simulations, we selected residues predicted to play critical roles in binding (Glu79 and Arg265 for ET; Glu81, Arg245, and Arg286 for MSX) for site-directed mutagenesis studies. The results showed that the Kd value of ET with the GLU79 mutant protein was 3.899 × 10^−5^ M (Figure 13e), and the Kd value with the ARG265 mutant protein was 1.346 × 10^−5^ M (Figure 13f); the Kd value with the ARG245 mutant protein was 1.886 × 10^−5^ M (Figure 13g), the Kd value of MSX with the GLU81 mutant protein was 1.214 × 10^−5^ M (Figure 13h), and the Kd value with the ARG286 mutant protein was 1.665 × 10^−5^ M (Figure 13i). These results indicate that the dissociation constants of the small molecules with the mutant proteins all increased, and the binding ability weakened.

### 3.7. Small-Molecule Inhibitors MSX and ET Reduced Fertilization Rate of Silkworm

**Figure 14:**
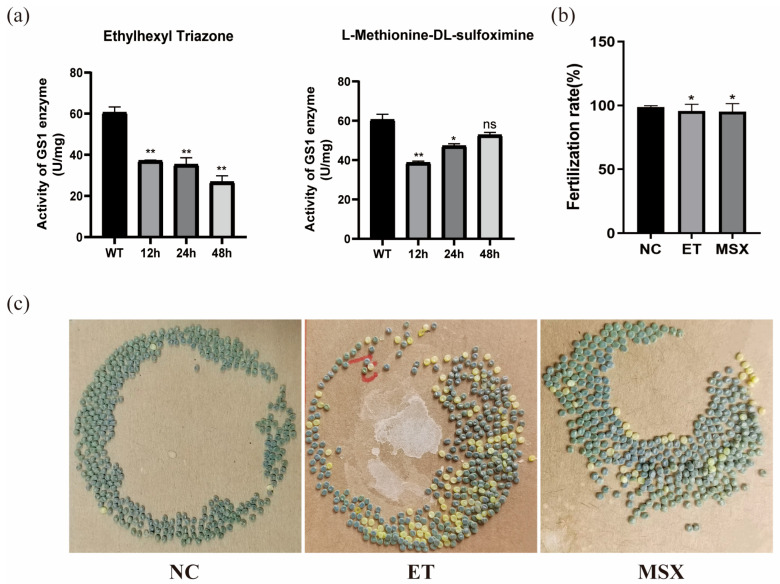
The effects of Ethylhexyl triazone (ET) and L-Methionine-DL-sulfoximine (MSX) on the fertilization rate of *Bombyx mori*. (**a**) Determination of enzyme activity in silkworm after the action of ET and MSX. (**b**) Statistical chart of Fertilization rate in silkworms. (**c**) The oviposition chart of the control group (NC) versus the oviposition chart of males treated with small molecules after mating with wild-type females. Three groups were repeated to take the mean, and the control group mean was used as the normalization factor; *n* = 3 independent experiments; *t*-test—ns means no significant difference, * indicates significance level *p* < 0.05, ** indicates significance level *p* < 0.01.

Male silkworms three days after pupation were injected with small molecules at a concentration of 2 μM. Enzyme activity was measured at 12 h, 24 h, and 48 h post-treatment. Compared to the DMSO-treated control, ET significantly reduced enzyme activity within 48 h, while MSX showed no significant effect at 48 h (Figure 14a). These results indicate that both small molecules have inhibitory effects on BmGS1, with ET demonstrating stronger inhibition.

Male moths treated with small-molecule compounds were paired with wild-type female moths for mating. After oviposition, the fertilization rates of the deposited eggs were analyzed. The results demonstrated that compared to natural mating pairs of male and female moths, the mating combinations of ET- or MSX-treated males with wild-type females exhibited a declining trend in fertilization rates post-oviposition (Figure 14b,c).

## 4. Discussion

In this study, we investigated the *glutamine synthetase 1* (*BmGS1*) gene in the model lepidopteran species *Bombyx mori*. Expression profiling of *BmGS1* via RT-PCR and qPCR revealed its abundant expression in the testis, with significantly higher transcript levels in sperm cells compared to the outer membrane tissue. During testis development, *BmGS1* was expressed throughout the fifth instar, pupal, and moth stages, peaking at the moth stage. These results suggest that BmGS1 may play a functional role in sperm development and maturation. Studies have revealed that glutamate serves as one of the essential amino acids required for sperm immobilization and storage in the murine epididymis, where it constitutes the most abundant free amino acid. The presence of glutamate likely plays a pivotal role in osmoregulation. During sperm maturation, the conversion of glutamate to glutamine significantly enhances sperm motility by eliminating excess glutamate [29,30]. The specific high expression of *BmGS1* in *Bombyx mori* spermatozoa suggests that GS may plays an important role in maintaining sperm function. However, due to differences in insect and mammalian reproductive systems, this could reflect functional convergence. Alternatively, it might be a conserved, homologous function across many species that is not yet clearly defined. Subcellular localization confirmed that BmGS1 is expressed in mitochondria and cytoplasm. The findings suggest that BmGS1 may also be functionally linked to mitochondrial metabolic processes. The glutamine synthesized by BmGS1 can be converted into α-ketoglutarate via the glutaminolysis pathway, subsequently participating in mitochondrial aerobic respiration and anabolic metabolism. This process not only provides cellular energy but also maintains redox homeostasis and protects cells from oxidative damage by scavenging reactive oxygen species [31]. We therefore hypothesize that BmGS1 may indirectly influence sperm quality by modulating mitochondrial physiological functions.

To elucidate the upstream regulatory mechanism of the *BmGS1* gene, we conducted a comprehensive analysis of the *BmGS1* promoter. First, we predicted the upstream regulatory factors of the *BmGS1* promoter. Based on the characteristic that *BmGS1* is highly preferentially expressed in sperm, we selected the reproduction-related transcription factors Bmdsx and Bmabd-A as candidate upstream regulatory factors. A dual-luciferase activity assay showed that Bmdsx had a promoting effect on the *BmGS1* promoter, while Bmabd-A had no significant effect. Furthermore, the EMSA experiment revealed that the transcription factor Bmdsx could specifically bind to the *BmGS1* promoter. The detection of changes in *BmGS1* expression after overexpression of *Bmdsx* at the cellular level showed that the expression level of the *BmGS1* gene was upregulated with the overexpression of *Bmdsx*. The detection of changes in *BmGS1* expression after interference with *Bmdsx* at the individual level showed that the expression level of the *BmGS1* gene was downregulated with the interference of *Bmdsx*. These results indicate that the *BmGS1* gene is positively regulated by the transcription factor Bmdsx. The regulation of *BmGS1* expression by reproduction-related transcription factors suggests that *BmGS1* plays an important role in spermatogenesis in *Bombyx mori*.

Potential small-molecule inhibitors (ET and MSX) against BmGS1 activity were screened using virtual screening, molecular docking, and MD simulation techniques. MD simulation results showed that the binding of the two small molecules to the protein remained stable throughout the simulation, with no significant displacement in the center of mass and relatively stable hydrogen bond counts. The van der Waals forces and electrostatic interactions during binding were also stable. Further analysis revealed that the amino acids GLU79 and ARG265 in the BmGS1 protein formed key hydrogen bond interactions with ET, while GLU81, ARG245, ARG286, and SER253 formed hydrogen bond interactions with MSX. These results not only confirm the binding stability of the inhibitors but also identify amino acid residues that may play critical roles in binding in the active site of BmGS1.

Fluorescence binding assays confirmed that the BmGS1 protein can bind to the small-molecule compounds ET and MSX in vitro. The binding sites of the small molecules to the protein were both located in the catalytic domain of the protein. Enzymatic activity assays also showed that both small molecules could inhibit the enzyme activity. Subsequently, based on the results of molecular dynamics simulations, five amino acids that may play critical roles in binding, Glu79, Arg265, Glu81, Arg245, and Arg286, were selected for site-directed mutagenesis. After specifically mutating these amino acid sites, the dissociation constants of both small molecules with the mutated target proteins increased, and the binding ability decreased, indicating that these amino acid sites may play an important role in the binding of small-molecule compounds to the BmGS1 protein. To investigate the effects of ET and MSX on BmGS1 protease activity, small-molecule administration experiments were conducted at the individual level of silkworms. Enzymatic activity assays showed that both ET and MSX treatments effectively reduced BmGS1 activity in the testes of silkworms. Additionally, results from small-molecule injection experiments revealed that when male moths treated with ET or MSX mated with wild-type female moths, the fertilization rate of the laid eggs exhibited a downward trend. It is important to note that this study has several limitations. First, the characterization of the inhibitors was performed qualitatively, and their half-maximal inhibitory concentration as well as dose–response relationships have not yet been determined, preventing the identification of their effective concentration range. Future work should address this through quantitative pharmacological experiments. Second, although the observed reduction in fertilization strongly correlates with BmGS1 inhibition, definitive proof of causality will require future direct sperm functional analyses. Finally, even though the inhibitors used in this study did not cause individual mortality, they may still exert certain effects on other organisms or the environment. Therefore, in considering their potential application as pest control agents, we must reasonably assess their specificity and environmental safety. Although this study only statistically analyzed the fertilization rate of the contemporary generation, and the observed downward trend was not significant—and future work could include statistics on fertilization rates across different reproductive cycles—this still preliminarily indicates that the identified small molecules ET and MSX can inhibit BmGS1 activity in silkworm testes, providing valuable clues and potential targets for the study of sperm activity inhibitors and male sterility.

In summary, we analyzed the expression profile, subcellular localization, and upstream regulatory mechanisms of the glutamine synthetase gene *BmGS1* in the silkworm. Two small-molecule inhibitors, ET and MSX, were screened and verified for their specific binding and inhibitory effects on BmGS1. Based on the functional conservation of GS1 across species (Figure S2), ET and MSX may exhibit broad application potential. Using the silkworm as a model organism, this study provides a foundational framework and conceptual basis for developing male sterility strategies in pest populations. In the future, such small-molecule inhibitors could be employed in sterility techniques targeting pests such as *Manduca sexta* or *Pectinophora gossypiella* by acting on orthologs of *BmGS1* in these species, thereby contributing to the development of more sustainable and environmentally friendly alternatives to broad-spectrum insecticides.

## Data Availability

The original contributions presented in this study are included in the article/supplementary material. Further inquiries can be directed to the corresponding author.

## Supplementary Materials

The following supporting information can be downloaded at https://www.mdpi.com/article/10.3390/insects17020135/s1, Figure S1: Protein purification; Figure S2: GS1 Protein Sequence Alignment Diagram; Table S1: Primers used in this study; Table S2: Ramachandran statistics; Table S3: Evaluation of libdockscore in the virtual screening of small-molecule inhibitors; Table S4: Per-residue energy contribution; Table S5: R^2^ values for the curves.
