# Supplementary material for: Glutamine Synthetase 1 Functions in Spermatogenesis in the Silkworm, Bombyx mori
Source: Insects. 2026 Jan 24;17(2):135. doi: 10.3390/insects17020135 (PMC12940822; doi:10.3390/insects17020135)
Supplement: Supplementary file 1 [file insects-17-00135-s001.zip › Figure S1.pdf]

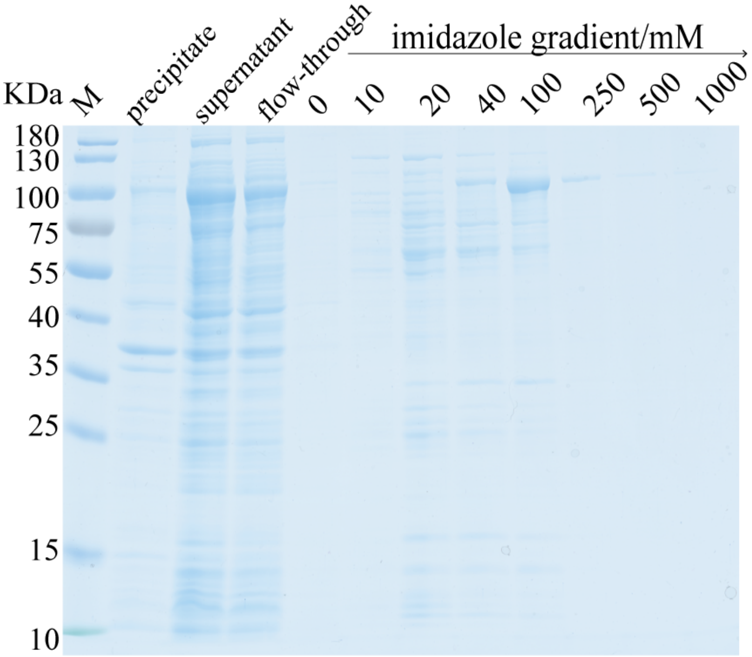

BmGS1-PCOLD-TF

Figure S1-A

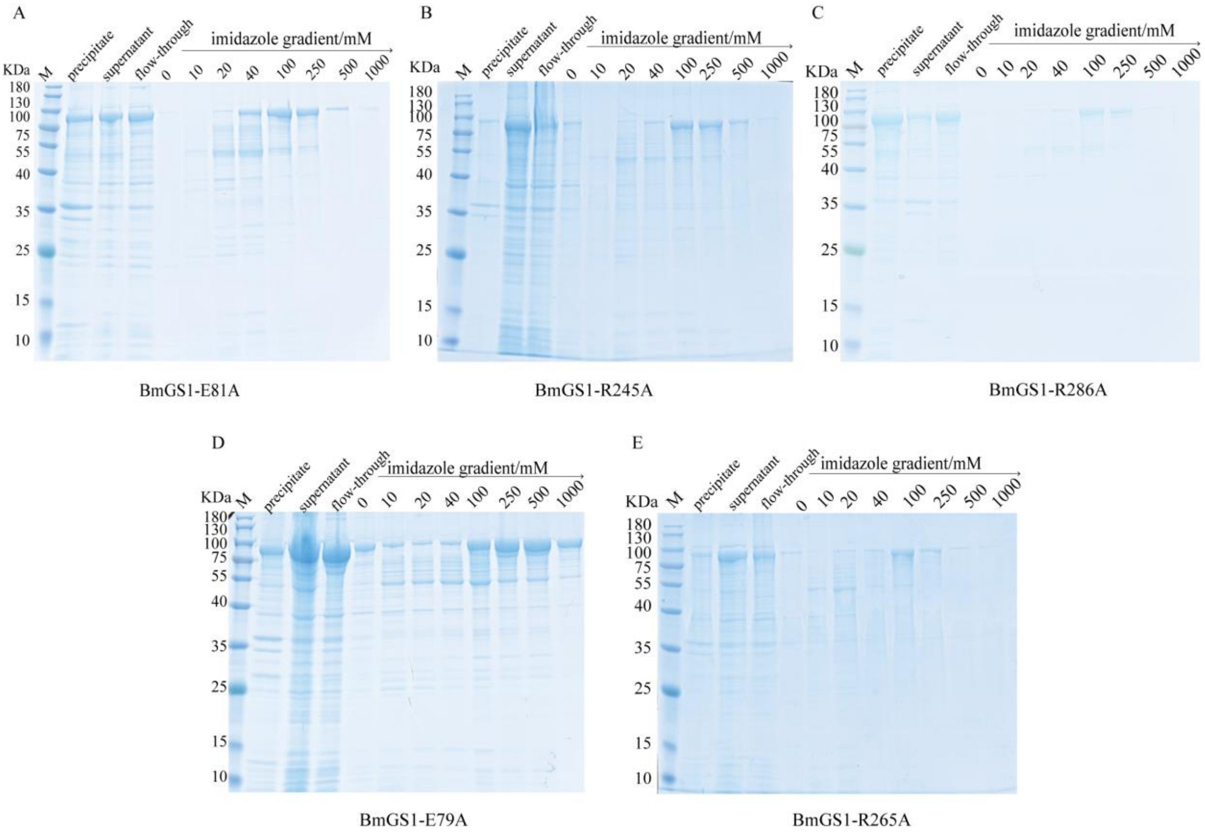

Figure S1-B

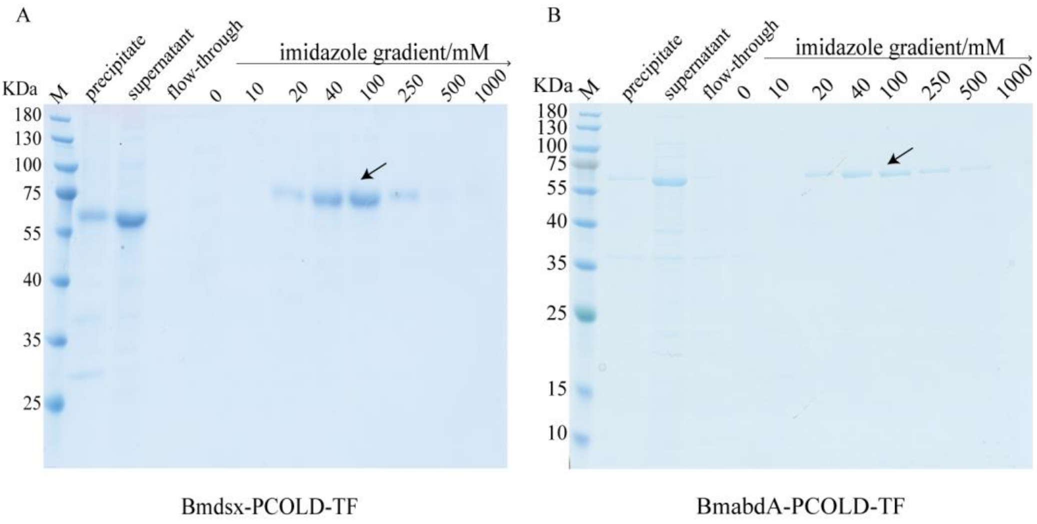

Figure S1-C

Figure S1. Protein purification. (**A**) BmGS1-PCOLD-TF protein purification; (**B**) Mutant protein purification. A represents the purification of BmGS1-E81A protein; B represents the purification of BmGS1-R245A protein ; C represents the purification of BmGS1-R286A protein; D represents the purification of BmGS1-E79A protein; E represents the purification of BmGS1-R265 protein; (**C**) Transcription factor Bmdsx and Bmabd-A protein purification.
