# Supplementary material for: Glutamine Synthetase 1 Functions in Spermatogenesis in the Silkworm, Bombyx mori
Source: Insects. 2026 Jan 24;17(2):135. doi: 10.3390/insects17020135 (PMC12940822; doi:10.3390/insects17020135)
Supplement: Supplementary file 1 [file insects-17-00135-s001.zip › Figure S2.pdf]

```
Bombyx mori                  ............................................................   0
Manduca sext                 ..........MTLLRYINRQPINKFFFRNLSLDNFTTGMSRVKSPINAYFNHYINLPIPE  50
Ostrinia furnacalis          ....MFSLQILNKTVGKCLHTTNIRKIRFLSQNCFTSGIAKLETPINAYYNHYIKLPIPE  56
Pectinophora gossypiella     ..................................MSSGI.RNEAPINAYYNHFLNLKIPA  25
Spodoptera litura            MYFKIINSVFTHRNKVLSKVFFKSHAVRNFSQENFSTGLTTTDRPINAYMFHYTNLKIPQ  60

Bombyx mori                  ..............................MVPKWSYDGSSTGQSDIKDSDITLLEVA  28
Manduca sext                 NKILATYVWIDGSGIHMRCKDRIINKEPCDISMVPKWSFDGSSTGQAKTNDSDTFLIESA 110
Ostrinia furnacalis          NKIFATYVWIDGSGIHMRSKDRIIDQIPCDIKMVPKWSFDGSSTGQATTDNSDTLLIESA 116
Pectinophora gossypiella     DKVFATYVWIDGSGINMRSKDRIINKVPHNISMIPKWSFDGSSTGQAKTENSDTLLIEAA  85
Spodoptera litura            NKIFATYVWIDGSGIHMRCKDRVIDKVPCNINMVPKWAFDGSSTGQARTEDSDTILLEVA 120

Bombyx mori                  VYRDPFRKSPHIIVLCETYSSEGLPTPTNHRVSSAITLSRICDQEPWFGIEQEYTMFDRD  88
Manduca sext                 IYRDPFRKSPHVIVLCETYYGDGTPTPTNHRAHCAQILSRISDQEPWFGIEQEYTMFDID 170
Ostrinia furnacalis          IYRDPFRKSPHIIVLCETYYGDGNPTPTNHRAHCAKVLSRITDQDPWFGIEQEYSIFDLD 176
Pectinophora gossypiella     MYRDPFRKSPNVIILCETYFDNGTPTDTNHRNHCAKVLSRISDKDPFFGIEQEYTMLDLD 145
Spodoptera litura            LYRDPIRKSPHVIVLCETYYGDGTPTPTNHRAHCANILSRLSDQEPWFGIEQEYTMFDVD 180

Bombyx mori                  HWFLGWPKPRGYP.DQDSKNSYCGVGEHVAGREIAECHARACIYSGMDYDGSNAEVMNGS 147
Manduca sext                 LWFLGWPKVRGYP.VTKSKYSYCGIGEHVAGRDIVECHTRACIYAGMDYEGSNAEVMKGS 229
Ostrinia furnacalis          LWFLGWPKMRGYP.VLKSKYSYCGMGEHVAGREVVECHARACVYSGMDYGGSNAEVMKGS 235
Pectinophora gossypiella     MWFLGWPKTRGFPSVGKNRFSYCGVGPYVVGREIAECHANACIYAGMDFEGTNAEVMKSA 205
Spodoptera litura            LWFLGWPKVRGFP.VIKSKYSYCGMGEHIAGREIAECHARACIYAGMDYGGSNAEVMKGS 239

Bombyx mori                  WEPQVGPTLGIKAADDIWIGRYLINRIAEHFDVIISFHPKPMGDGHAGIGCHHNFSTKKM 207
Manduca sext                 WEPQVGTTMGVKAADDLWLGRFLINRIAEKFGVIISYHPKPVGKNQPGIGCHHNFSVKKM 289
Ostrinia furnacalis          WEPQVGTTPGIKAADDLWVGRYLIGRIAENFGVIISYHPKPMGKDQPGIGCHHNFSVNKM 295
Pectinophora gossypiella     WEPQVGIAKGIKAADDIWLGRYLITRIAEYFGATISFHPKPMGKYIPGTGCHHNFSIKKM 265
Spodoptera litura            WEYQVGTTVGIKAADDLWLGRYLIGRIAENFGIIISYHPKPVGKNQPGIGCHHNFSTKKM 299

Bombyx mori                  RADQGVTEIKRICKILGDQHENIIKSYDLKDGEDNKKRLVGKPETSSYDSGRVGISDRSA 267
Manduca sext                 RCDGGIKEIEKVCKILGDQHDKLIKNYGLGNGEENKKRLTGKPETASYLSGRWGVADRGA 349
Ostrinia furnacalis          RCDDGVKEIERVCKLLGEKHKTYMKSYGLGDGEENRKRLTGKPETASFDTGRWGVADRGA 355
Pectinophora gossypiella     RSEGGIEEVQRVCKILGEKHDTYIKNYGLGNGEENKIRLTGKHETAPYDSGRWAVADRGA 325
Spodoptera litura            RDEGGITEIERVCKVLGAKHGDYMKHYGLGDGQDNRKRLTGKPETASFDVGKWAIADRTA 359

Bombyx mori                  SIRIQKKVISEGKGYFEDRRPAGNCDPYMVCGLIAKTCI                      306
Manduca sext                 SVRLQKSVVSEGKGFLEDRRPAGDCDPYRVCAFIAETCL                      388
Ostrinia furnacalis          SIRLQRSVVTKGKGFLEDRRPAGDCDPYRVCALIAETCS                      394
Pectinophora gossypiella     SIRIQRSTVINKKGFLEDRRPAGDCDPYRVCALIAETCL                      364
Spodoptera litura            SIRLQRSVKTKKKGFLEDRRPAGDCDPYRVCALIAETCV                      398
```

Figure S2. Multiple sequence alignment of GS1 proteins from several lepidopteran insects.
