# Supplementary material for: Glutamine Synthetase 1 Functions in Spermatogenesis in the Silkworm, Bombyx mori
Source: Insects. 2026 Jan 24;17(2):135. doi: 10.3390/insects17020135 (PMC12940822; doi:10.3390/insects17020135)
Supplement: Supplementary file 1 [file insects-17-00135-s001.zip › Table S1.pdf]

Table S1. Primers used in this study.

Name	Primer sequence	Purpose
Gs1KL-F	ATGGTGCCGAAATGGTCGTATGA	Gene clone and RT-PCR
Gs1KL-R	TCAAATACATGTTTTAGCAATTAAG	
Gs1-q-F	TCCTAAGCCAATGGGAGATG	
Gs1-q-R	CAGAAATGCCTACACGACAC	
Actin3-F	AACACCCCGTCCTGCTCACTG	qPCR
Actin3-R	GGGCGAGACGTGTGATTTCCT	
sw-F	TTCGTACTGGCTCTTCTCGT	
sw-R	CAAAGTTGATAGCAATTCCCT	
1180-F	ATAACGAAGCTGTGCAATAG	Sequencing primers
1180-R	CAACTGTTGGGAAGGGCGAT	
GS1-1180EGFP-F	**GACGAGCTGTACAAG**GGATCCATGGTG CCGAAATGGTCGTATGA	Subcellular localization amplification primers
GS1-1180EGFP-R	**CTGATTATGATCTAGAGTC***GCGGCCGC*TC AAATACATGTTTTAGCAATTAAG	
GS1-pctf-F	**GAAGGTAGGCATATG**GAGCTCATGGTG CCGAAATGGTCGTATGA	Prokaryotic expression vector construction
GS1-pctf-R	**AGACTGCAGGTCGAC**AAGCTTTCAAAT ACATGTTTTAGCAATTAAG	
PCOLD-F	ACGAGCTGATGAACCAGCAGGCG	Sequencing primers
PCOLD-R	CGCGATCGATTATTTATTTCCTG ATTCC	
Glu79Ala-F	TGGTTTGGTATAGCACAAGAATACACAA	
Glu79Ala-R	GTGCTATACCAAACCATGGTTCTTGGTC	
Arg265Ala-F	GGCATTTCTGATGCAAGTGCGTCGATAC	
Arg265Ala-R	TTGCATCAGAAATGCCTACACGACACGA	
Arg286Ala-F	TACTTTGAAGATGCAAGACCTGCAGGCA	Site-directed mutagenesis vector construction
Arg286Ala-R	TTGCATCTTCAAAGTATCCTTTGCCTTC	
Glu81Ala-F	GGTATAGAACAAGCATACACAATGTTCG	
Glu81Ala-R	ATGCTTGTTCTATACCAAACCATGGTTC	
Arg245Ala-F	AAGAAGGCGTTGGTAGGAAAAT	
Arg245Ala-R-5	TCCTACCAACGCCTTCTTGTTATCT	
promoter-F	CAACACATATCACTTGGTGTTTG	Promoter cloning
promoter-R	TTCTAAATCACAAGGCTCC	
promoter-pGL3-F	**GCGTGCTAGCCCGGG**CTCGAGCAACAC	Promoter expression

	ATATCACTTGGTGTTTG	vector construction
promoter-pGL3-R	**CAGTACCGGAATGCC**AAGCTTTTCTAAATCACAAGGC	
abd-A-KL-F	ATGCGAAGGCGAGGACGACAAAC	
abd-A-KL-R	TTACACGGCTCGTAGTTCTTTCTTT	
abd-A-domain-F	ATGAGTTCCAAGTTCATCATCGA	Transcription factor amplification primers
abd-A-domain-R	TTACGTGGGGACCTTGTTCACTTTG	
dsxKL-F	ATGGTGTCGATGGGCTCATGGAAAC	
dsxKL-R	GGTATCAGTGGTGGCATGGTTGGCG	
abd-A-1180-F	**TGTTAGAGGATTGGT**GGATCCATGCGAAGGCGAGGACGACAAAC	
abd-A-1180-R	**CTGATTATGATCTAGAGTC**GCGGCCGCTTACACGGCTCGTAGTTCTTTCTTT	
1180Damain-dsx-F	**TGTTAGAGGATTGGT**GGATCCATGGATTACAAGGATGACGACGATAAGGCCCCTCCTAACTGTGCG	Transcription factor vector construction
1180Damain-dsx-R	**CTGATTATGATCTAGAGTC**GCGGCCGCTCACTGGGCCCGTCTGATTGCCGTTTGC	
Dsx-pctf-F	**GAAGGTAGGCATATG**GAGCTCATGGTGTCGATGGGCTCATGGAAAC	
Dsx-pctf-R	**AGACTGCAGGTCGAC**AAGCTTGGTATCAGTGGTGGCATGGTTGGCG	Prokaryotic expression vector construction
abdA-pctf-F	**GAAGGTAGGCATATG**GAGCTCATGCGAAGGCGAGGACGACAAACT	
abdA-pctf-R	**AGACTGCAGGTCGAC**AAGCTTTTACACGGCTCGTAGTTCTTTCTTT	
dsx-q-F	GGATGGAGTTGGATAGGCCT	Sequencing primers
dsx-q-R	GGTATCAGTGGTGGCATGGT	
abd-A-Probe-F	biotin- ATTATATTATTAATTATTACTGTAC	
abd-A-Probe-R	GTACAGTAATAATTAATAATATAAT	
dsx-Probe-F	biotin- ATTTTTTAAACAATGTACTGCTTTA	Probe sequence
dsx-Probe-R	TAAAGCAGTACATTGTTTAAAAAAT	
dsx-cold-Probe-F	ATTTTTTAAACAATGTACTGCTTTA	
dsx-cold-Probe-R	TAAAGCAGTACATTGTTTAAAAAAT	

Mut-dsx-Probe-F	CGGGGGGCCCACCGTGCAGTAGGGC
Mut-dsx-Probe-R	GCCCTACTGCACGGTGGGCCCCCCG

Note: Underlined sequences indicate restriction enzyme sites, red-colored bases denote mutated nucleotides, and bolded regions represent homology arms.
